# Decentralized Immunization Monitoring: Lessons Learnt from a Pilot Implementation in Kumbotso LGA, Kano State, Nigeria

**DOI:** 10.3390/vaccines13070664

**Published:** 2025-06-20

**Authors:** Adam Attahiru, Yahaya Mohammed, Fiyidi Mikailu, Hyelshilni Waziri, Ndadilnasiya Endie Waziri, Mustapha Tukur, Bashir Sunusi, Mohammed Nasir Mahmoud, Nancy Vollmer, William Vargas, Yusuf Yusufari, Gustavo Corrêa, Heidi W. Reynolds, Teemar Fisseha, Talatu Buba Bello, Moreen Kamateeka, Adefisoye Oluwaseun Adewole, Musa Bello, Imam Wada Bello, Sulaiman Etamesor, Joseph J. Valadez, Patrick Nguku

**Affiliations:** 1 African Field Epidemiology Network (AFENET), Abuja 900231, Nigeria; ymohammed@afenet.net (Y.M.); fmikailu@afenet.net (F.M.); hwaziri@afenet.net (H.W.); ewaziri@afenet.net (N.E.W.); mtukur@afenet.net (M.T.); taallybello@yahoo.com (T.B.B.); mkamateeka@afenet.net (M.K.); aadewole@afenet.net (A.O.A.); pnguku@afenet.net (P.N.); 2 Kano State Primary Health Care Development Agency, Kano 700101, Nigeria; mbs966@yahoo.com (B.S.); mnasirmahmoud@ksphcmb.org (M.N.M.); imamsharfadi@gmail.com (I.W.B.); 3 John Snow Incorporated (JSI), Arlington, VA 22209, USA; nancy_vollmer@jsi.com (N.V.); teemar_fisseha@jsi.com (T.F.); 4 Liverpool School of Tropical Medicine, Liverpool L3 5QA, UK; willivargasv@gmail.com (W.V.); joseph.valadez@lstmed.ac.uk (J.J.V.); 5 Gates Foundation, Seattle, WA 98109, USA; yusuf.yusufari@gatesfoundation.org; 6 Gavi, the Vaccine Alliance, 1218 Geneva, Switzerland; gcorrea@gavi.org (G.C.); hreynolds@gavi.org (H.W.R.); 7 African Health Budget Network (AHBN), Abuja 900108, Nigeria; mmbello.cmed@buk.edu.ng; 8 National Primary Health Care Development Agency, Abuja 900288, Nigeria; sulaiman.etamesor@nphcda.gov.ng

**Keywords:** zero dose children, vaccination coverage, vaccine hesitancy, social determinants of immunization, service delivery, lot quality assurance sampling, immunization surveys

## Abstract

Background: Immunization coverage in Nigeria is low, with many children missing out on important lifesaving vaccines. To enable a better understanding of contextual factors towards increasing uptake, we piloted a Decentralized Immunization Monitoring (DIM) approach in the Kumbotso local government area (LGA) of Kano state, Nigeria, to identify wards with low vaccination rates and understand why this is happening. The findings were used to improve routine immunization (RI) programs and reduce the number of unvaccinated children and children yet to receive their first dose of diphtheria–pertussis–tetanus (DPT) vaccine, referred to as Zero-Dose children (ZD). Methods: This study adopted a cross-sectional design approach using the Behavioural and Social Drivers of Vaccination (BeSD) framework and the Lot Quality Assurance Sampling (LQAS). The study population comprised caregivers of children aged 0–11 months and 12–23 months across the 11 wards in Kumbotso District, Kano State, Nigeria, using a segmentation sampling approach. The study covered 209 settlements selected using probability proportionate to size (PPS) sampling from the wards. Univariate and bivariate analyses were performed to show patterns and relations across variables. Results: Out of 418 caregivers surveyed, 98.1% were female. Delayed vaccination was experienced by 21.9% of children aged 4.5–11 months, while the prevalence of ZD was estimated at 26.8% amongst the older cohort (12–23 months). A total of 71.4% of the delayed group and 89.1% of the ZD group remained unvaccinated. Caregiver education, rural residence, and home births correlated with delayed/ZD status (*p* < 0.05). Logistic regression associated higher caregiver education with reduced delayed vaccination odds (OR:0.34, *p* < 0.001) and urban residence with lower ZD odds (OR:1.89, *p* = 0.036). The antigen coverages of BCG (81.5%), DPT3 (63.6%), and measles 1 (59.7%) all surpassed the national dropout thresholds. Kumbotso, Unguwar Rimi, and Kureken Sani wards were all identified as underperforming and therefore targeted for intervention. Negative vaccine perceptions (50% delayed, 53.6% ZD) and distrust in health workers (46.4% delayed, 48.2% ZD) were significant barriers, though the caregiver intent to vaccinate was protective (OR: 0.27, *p* < 0.001). The cost of accessing immunization services appeared to have a minor effect on coverage, as the majority of caregivers of delayed and ZD children reported spending less than 200 Naira (equivalent to USD 0.15) on transport. Conclusions: This pilot study highlighted the utility of LQAS and BeSD in identifying low-performing wards, barriers, and routine immunization gaps. Barriers included low caregiver education, rural residence, and negative vaccine perceptions/safety. Caregiver education and urban residence were protective factors against delayed and ZD vaccination, suggesting social and systemic barriers, particularly in rural and less educated populations. Antigen-specific coverage showed disparities, with dropouts for multi-dose vaccines exceeding the national thresholds of 10%. Targeted measures addressing education, trust, and systemic issues are needed. Findings emphasize decentralized monitoring, community engagement, and context-specific strategies to reduce ZD children and ensure equitable vaccination in Nigeria.

## 1. Introduction

Routine immunization is a cornerstone of public health, providing a systematic approach to delivering lifesaving vaccines and maintaining population immunity. In Nigeria, the coverage of immunization has remained suboptimal, accounting for the highest number of children globally yet to receive the first dose of diphtheria, pertussis, and tetanus (DPT) vaccine. The national coverage for the third dose of the DPT vaccine was reported at 33% in 2016, with a modest increase to 57% by 2021 [1] and to 53% in 2023, based on the National Demographic and Health Survey (NDHS) [2,3].

The Routine Immunization Technical Working Group (RITWG), in line with the Immunization Agenda 2030 [4] and Gavi, the Vaccine Alliance 5.0 strategy [5], has developed a National Strategic Plan comprising innovative strategies such as the zero-dose reduction plan; identify, enumerate, and vaccinate; and optimized outreach services, all aimed at reducing the population of zero-dose children to 15% by 2024 [6]. These innovative strategies inculcate adaptive learning processes around equity, partnership, community ownership, and program integration and align with the ‘identify’ and ‘reach’ components of the Identify–Reach–Monitor–Measure–Advocacy (IRMMA) framework [3]. According to the Gavi operational definition, a child is considered as “zero dose” (ZD) if they have not received the first dose of DPT or DPT-containing vaccines [7,8].

The Gavi-funded Nigeria Zero Dose Country Learning Hub (CLH) adopted the Decentralized Immunization Monitoring (DIM) approach to provide timelier qualitative and quantitative evidence from communities to synthesize learnings for prompt, data-driven decision-making at the local government area (LGA) and ward levels. The CLH target areas include eight LGAs in four selected states, namely Kano (Kumbosto and Sumaila LGAs), Bauchi (Bauchi and Ganjuwa LGAs), Borno (Maiduguri and Jere LGAs), and Sokoto (Wamakko and Tambuwal LGAs). The DIM aims to yield vaccination coverage data and contextual information to help inform the refinement/redesign of routine immunization interventions at the grassroots levels by highlighting the existing facilitators and barriers hindering routine immunization (RI) and the journey to vaccination by caregivers.

This study presents the results of the pilot application of the DIM in Kumbotso LGA of Kano State. Results were used to refine the DIM approach prior to scaling up in the remaining seven selected LGAs.

## 2. Objective

This study aims to assess the RI performance at the ward level, understand local factors influencing vaccination uptake, and estimate the LGA-level coverage for key immunization indicators. This approach generates evidence to support the State Primary Health Care Management Board (SPHCMB) in refining its immunization policies and programs.

## 3. Materials and Methods

A cross-sectional design consisting of a household (HH) survey in all wards was adopted. Households were selected using Lot Quality Assurance Sampling (LQAS) [9]. The classic LQAS methodology adopted the ward as the supervision area (SA) and the LGA as the catchment area (CA). The vaccination status of surveyed children was primarily parent-held child health cards. Where unavailable, caregiver recall was accepted and recorded accordingly.

**Figure 1:**
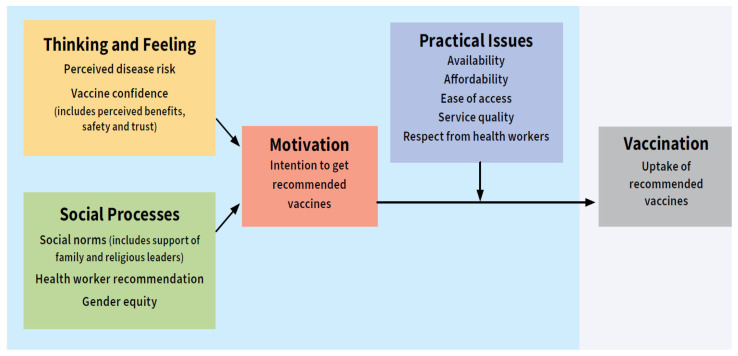
Behavioural and Social Drivers of Vaccination Framework.

The WHO Behavioural and Social Drivers (BeSD) of Vaccination framework [10] was adopted to understand contextual drivers and facilitators of childhood vaccination across five domains (Figure 1).

**Figure 2:**
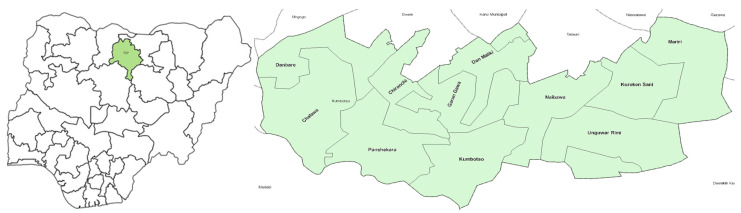
Map of Nigeria showing Kano State and Kumbotso, Nigeria.

Study Area: Kumbotso, an LGA situated in Kano State, Nigeria (Figure 2), spans 2593 km^2^ and is home to approximately 484,200 residents [11]. In the LGA, there are a total of 33 public and 23 private health facilities. Notably, Kumbotso was identified as the most important amongst the 15 priority LGAs for numbers of zero-dose children, with an estimated count of 12,650 ZD children [11].

Study Sites: The study was implemented in Kumbotso LGA of Kano State [12]. All 11 wards in the LGAs were included.

Study Population: Caregivers of two cohorts of children were sampled, namely children aged 0 to 11 months and 12–23 months. Newly relocated residents, visitors with less than three months, and secondary caregivers without history or evidence of vaccination were excluded.

Sampling Technique and Sample Size: A multi-staged sampling approach was adopted. The LQAS approach was used for assessing program performance and monitoring, with the LGA adopted as the catchment area (CA) and the wards as supervision areas (SA) [9,12,13].

- From the Masterlist of settlements (sampling frame), 19 interview locations were identified using probability proportionate to estimated size (PPES) in each of the 11 wards (totaling 209 interview locations).
- Selected interview locations were segmented using sketched maps with an estimated number of households. To identify reference and starting compounds, one segment was randomly selected using a table of random numbers [14], and a household within the selected segment number was randomly selected.
- In each of the selected households, one eligible caregiver (0–11 months or 12–23 months) was selected and interviewed. The next closest house was visited to sample caregivers of the remaining cohort [15].
- This process was systematically repeated at each of the selected interview locations.

Instrument: The BeSD standardized questionnaire was adopted [10] to document factors contributing to low vaccination uptake, barriers to vaccination, socio-demographic characteristics, and vaccination history of sampled children. A questionnaire control sheet was developed to document reasons for non-eligibility. A field team performance and validation tool was developed to ensure reliability of data collected.

Data Management:

- Training: A total of 22 research assistants and 8 supervisors were recruited and trained for two days using an adult-learning approach to enhance understanding of the research methodology, tools, and interview techniques. It included both field practicum and classroom role play.
- Data Collection: Field data collection was conducted over six days in April 2024, across 209 interview locations, with research assistants handling an average of three settlements per day.
- Quality Control: All data were collected electronically on smartphones using Open Data Kit (ODK) [16]. Quality control measures such as skip patterns and constraints were embedded in the tools developed. Daily data review and feedback by a Data Manager was institutionalized.
- Data Security: All collected data were securely transmitted to a cloud-based ODK Central server, with multiple layers of access authentication to ensure data protection.

Ethical Approval: Ethical approval was received from the Kano State Health Research and Ethics Committee (HREC) with the HREC Protocol Number: SHREC/2023/4271. Written informed consent was obtained from all study participants.

Data Analysis: Data cleaning and analysis were conducted using Microsoft Excel and R-Studio (using tidyverse—version 1.3.1 and gtsummary packages—version 1.7.2). Exploratory analysis was conducted to understand and summarize demographic and behavioral characteristics. Bivariate analyses adopted chi-square, Fisher exact, and Z-tests (where appropriate) to explore associations between categorical variables with statistical significance set at a 95% confidence level. Logistic regression analysis was employed to identify predictors of vaccine uptake. Variables found to be statistically significant in bivariate analysis (*p* < 0.05) were included in the multivariate model using stepwise regression. Logistic regression was adopted due to its ability to model relationships with multiple independent variables and a binary outcome while adjusting for confounders. To examine timeliness of vaccination, a subsample of children aged 18 weeks (4.5 months) to 11 months were selected following recommendations of Correa et al. [17]. Timeliness was categorized as either ‘delayed’ (no DPT1) or ‘undelayed’ (received DPT), while the older cohort (12–23 months) was categorized into ZD and non-ZD. A weighted, antigen-specific coverage analysis was conducted to estimate coverage at the LGA level. Ward categorization was based on estimated LGA weighted coverage and the national target of 80%. A 95% confidence interval (alpha error of 0.05) and a statistical power of at least 90% (beta error of <10%) were adopted for reliability [4,18]. Wards with coverage below these thresholds were classified as underperforming.

## 4. Results

Across the 11 wards of Kumbotso, a total of 418 respondents were surveyed; 50% (209) were caregivers to children aged 0–11 months and 50% to children aged 12–23 months. A significant majority of caregivers, 410 (98.1%), were females and were the primary caregivers. Over a third of caregivers had no formal education, and 306 (73.20%) of them fell within the 1st and 3rd quintiles using the Nigeria Equity Tools [19] comprising 10 household items (Table 1).

In the study sample of 418 children, 72 (17.2%) were reported to belong to the no immunization category. The prevalence of ‘no immunization’ was higher among children aged 12–23 months, with 41 (19.6%) unvaccinated, compared to 31 (14.8%) in the 0–11-month cohort. Additionally, within the younger cohort, over 65% of the unvaccinated cases were older than four and a half months, indicating a significant delay in receiving their initial vaccinations.

### 4.1. Demographic and Socioeconomic Factors Affecting the Uptake of Immunization

This study explores the association between caregiver characteristics, including education, income, and place of residence, and child outcomes related to vaccination timeliness and status for 4.5–11-month and 12–23-month age groups (Table 2). Understanding these underlying factors in the Kumbotso LGA will influence immunization strategy implementation while establishing a baseline for program monitoring and performance.

The analysis revealed demographic and sociodemographic factors influencing vaccination delays and ZD among children. ZD children were significantly more likely to be present in rural areas than in urban areas. Low or no educational status was found to be associated with delayed vaccination and ZD status in both 4.5–11-month (*p*-value of 0.003) and 12-23-month (*p*-value of <0.001) age groups. Additionally, poorer households were found to be significantly associated with delayed vaccination and ZD status in the 4.5–11-month (*p*-value of 0.034) and 12–23-month (*p*-value of 0.010) age groups. Home delivery and delivery with a traditional birth attendant were found to be significantly associated with delayed (No DPT) vaccination in the 4.5-11-month (*p*-value of 0.008) age groups as well as ZD status in the 12–23-month (*p*-value of <0.001) age groups.

A logistic regression analysis was conducted to identify the sociodemographic factors associated with delayed vaccination among caregivers (Table 3). Low educational level was found to be significantly associated with delayed vaccination (OR: 0.34, 95% CI: 0.22–0.53, *p*-value: <0.001). Specifically, higher educational levels were associated with a lower likelihood of delayed vaccination. In contrast, neither wealth index (OR:0.88, 95% CI: 0.71–1.09, *p*-value = 0.235) nor place of delivery (OR: 0.79, 95% CI: 0.54–1.16, *p*-value = 0.229) were significantly associated with delayed vaccination in this model.

The result from the logistic regression shows a significant negative relationship between the caregiver educational level and ZD status (Table 4) with an OR of 0.37, 95% CI of 0.24–0.58, and a *p*-value of <0.0001; the results suggest that caregivers with higher levels of education are less likely to have ZD children compared to those with lower educational levels. There is a significant association between settings and ZD status with an OR of 1.89, 95% CI of 1.04–3.44, and a *p*-value of 0.0365; the results suggest that caregivers in urban areas are less likely to have ZD children compared to those in rural locations. Wealth index and point of delivery are not significantly associated with ZD status in the model and do not appear to have a statistically significant impact on ZD status with an OR of 0.87 and 95% CI of 0.70–1.07 and an OR of 0.85 and 95% CI of 0.58–1.25, respectively.

#### 4.1.1. Vaccination Uptake and Coverage

The vaccination timeliness was evaluated among the younger cohort (4.5–11 months) as a key performance indicator for immunization program effectiveness. The analysis revealed that 28 (21.9%) of the 128 eligible children experienced a delayed uptake of DPT1. Twenty (71.4%) of the twenty-eight children with delayed uptake remained completely without any form of immunization (no immunization). Among the undelayed group, defined as those with evidence of receiving at least one dose of DPT1, 81 (81.0%) of the eligible children were found to have received DPT3. This means that 19.0% had an incomplete vaccination. High vaccination card retention was observed at 90.0% among the undelayed group, indicating better adherence to immunization schedules and potentially greater caregiver awareness and commitment to completing the recommended vaccination series. The reasons for non-availability of cards were reported to include misplacement of cards.

For children aged 12–23 months (Table 5), Kumbotso showed a strong immunization initiation, with 168 (80.4%) receiving at least one dose of immunization of any antigen. Card retention amongst this group of ever-vaccinated children was high (85.7%). ZD was, however, estimated at 26.8%, with 41 (89.1%) of the 56 children with no immunization. Amongst the non-zero dose category, Bacillus Calmette–Guérin (BCG) had the highest coverage of 81.5%, while other birth dose antigens had lower coverage, including hepatitis B0 and oral polio virus vaccine at 71.2% and 77.9%, respectively. DPT coverage was estimated at 75.5% for dose 1 and at 63.6% for dose 3, indicating good access to immunization services but suboptimal utilization. The dropout rate increased with child age, suggesting challenges with retention. The proportion of under-immunized was estimated at 18.3%. The dropout rate among multidose vaccines was observed to exceed the national threshold of 10%, reaching 12% for DPT (1 and 3) and PCV (1 and 3) and 15.2% for IPV (1 and 2).

#### 4.1.2. Ward-Level RI Performance

Children aged 12–23 months were used in the categorization of wards into priority and non-priority areas, based on the LQAS approach using the decision rules table [20]. Ward-level performance was evaluated using the standardized LQAS table showing a decision threshold against coverages across each of the selected immunization antigens. Wards were classified based on whether the number of children who received the antigen met the predefined decision rules, which were derived from the weighted average coverage for each antigen at the LGA level.

When compared to the LGA’s average antigen coverage, three wards—Kumbotso, Unguwar Rimi, and Kureken Sani—demonstrated consistently low performance across most antigens. Six wards—Challawa, Gurin Gawa, Mariri, Naibawa, and Pansheka—achieved acceptable performance across all antigens. Further analysis comparing ward-level performance to the national target of 80% (with a decision rule of 13) revealed that only three wards—Gurin Gawa, Naibawa, and Panshekara—met the target for all the antigens. The majority of the wards with sub-optimal performance, however, had five key antigens, DPT2, DPT3, IPV2, PCV2, and PCV3, not meeting the national target. Notably, all wards except Gurin Gawa, Naibawa, and Pansheka failed to meet the Measles 1 coverage, underscoring the need to intensify retention and follow-up to improve vaccination completion.

### 4.2. Behavioral and Social Drivers of Vaccination Results

To gain a deeper understanding of the complex interplay between individual perceptions, community norms, and systemic factors, it is necessary to explore the behavioral and social drivers influencing vaccination uptake. This will facilitate the development of a tailored strategy. Across the four [4] domains—thinking and feeling, social processes, motivation to vaccinate, and practical issues—this study attempts to identify the factors affecting uptake in Kumbotso LGA while comparing across the two age cohorts (Table 6).

Thinking and Feeling: Caregivers’ decisions about childhood vaccination are shaped by a combination of cognitive and emotional factors, where their thoughts influence perceptions, and emotions such as fear and responsibility play a significant role in decision-making. In Kumbotso LGA, vaccine importance, vaccine safety, and trust in healthcare workers were found to be significantly associated with vaccination uptake across the two age cohorts. The analysis revealed that about 50.0% of caregivers (*p*-value of <0.001) in the delayed category and 53.6% (*p*-value of <0.001) in the ZD category held negative perceptions on vaccine importance, while positive perceptions were noted in over 95% of caregivers in the undelayed and NZD categories.

Social Processes: Social processes play an important role in shaping health behaviors, including vaccination uptake. The BeSD framework explores how religious and community leaders, close friends, family members, and parents influence caregivers’ attitudes towards vaccination. The analysis showed that support from close friends and family significantly impacts caregivers’ decisions to vaccinate across both age cohorts, at a *p*-value of 0.007 for the delayed group in the 4.5–11-month-old cohort and a *p*-value of <0.001 for the ZD in the 12–23-month age group, with peer opinions particularly influencing the decision amongst the younger age cohort at a *p*-value of 0.041. Gender dynamics were found to be evident, as needing permission to vaccinate was significantly associated with ZD status (*p* = 0.019), highlighting the influence of gender roles and decision-making within households.

Motivation: Caregivers’ intention to vaccinate is an important determinant to improving vaccination uptake and is influenced by multiple factors. The analysis reveals a strong relationship between caregiver intention to vaccinate their children and uptake of vaccination at a *p*-value of <0.001 for both age cohorts. A total of 21 (75.0%) caregivers in the delayed category of the younger cohort and 39 (69.6%) of the ZD caregivers category reported having the intention to vaccinate their children with some or none of the recommended antigens. The analysis highlighted that a substantial percentage of caregivers in both groups (25.0% amongst the delayed group and 30.4% amongst the 12–23-month group), despite having the intention to vaccinate their children with all recommended antigens, remained either delayed or ZD. Factors such as the availability and adequacy of vaccines at health facilities, spousal support, and the provision of financial or non-financial incentives, including soap, mosquito nets, and milk, were identified as key motivators.

Practical Issues: Understanding the contextual barriers and facilitators to vaccination, such as the associated cost of vaccination, availability of service, knowledge of where to vaccinate, and quality of care, is important in improving the vaccination coverage. In Kumbotso LGA, the findings showed that service satisfaction and ease of payment (affordability) for vaccination are important practical determinants of vaccination for the older cohort. The finding shows that over 93.9% of the undelayed and NZD caregivers reported satisfactory experience of care compared to 78.8% of delayed and ZD caregivers, who reported moderate to high service satisfaction. However, these findings in the younger cohort were not statistically significant, while they were significant at a *p*-value of 0.047 in the older cohort. Reasons for dissatisfaction included disrespect by healthcare workers, long waiting hours, and vaccine unavailability. Additionally, knowledge of where to vaccinate children was identified as a significant factor (*p*-value of <0.001) using the Z test, with over 95% of caregivers in both age cohorts being aware of the appropriate health facility for childhood vaccination. About 80.4% of caregivers across both age cohorts found it very or moderately easy to pay for the costs associated with vaccination. However, a higher proportion of caregivers in the 12-23-month cohort (22.0%) reported payment challenges compared to those in the 4.5–11-month cohort (15.6%). The findings were statistically significant for the older age cohorts at a *p*-value of <0.001.

**Figure 3:**
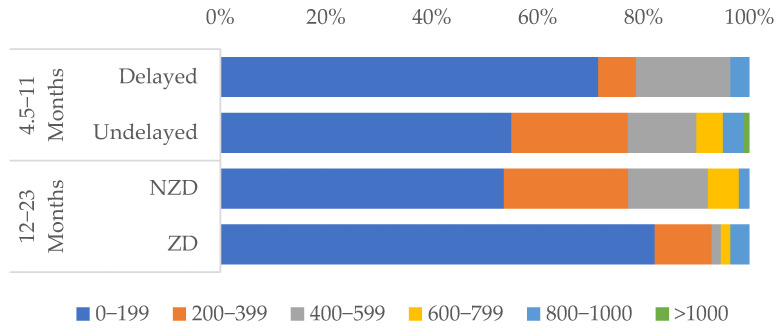
Bar chart showing caregivers’ reports of the cost of round trip to RI facility, categorized by immunization status.

Further analysis revealed that about 70% of delayed vaccinations and 80% of ZD (Figure 3) reported spending less than 200.00 (1 United States Dollar = 1302.67 Nigerian Naira as of 1st April 2024) [21] to reach an RI facility, and over 97% of caregivers had knowledge of where to vaccinate their children, suggesting close geographic proximity to healthcare services.

## 5. Discussion

The DIM approach provided a robust framework for mapping the contextual drivers of vaccination uptake. The Lot Quality Assurance Sampling (LQAS) method was found to be operationally feasible and cost-efficient in the study area. The approach yielded timely, actionable data that enabled the identification of poorly performing wards and provided reliable estimates of immunization coverage at the LGA level while also uncovering key socioeconomic and behavioral determinants of ZD and delayed immunization. The evidence generated is crucial to decision and policy makers in guiding targeted interventions and refinement of existing immunization strategies to improve equity and program effectiveness.

The ward-level RI performance revealed important information about the state of immunization coverage and the factors influencing it. This study provides an analysis of the immunization performance, offering demographic and vaccination uptake data that give a clear picture of the RI landscape. The predominant married females align with the cultural context of northern Nigeria, where they remain the primary caregivers [2]. Unvaccinated children were more among the 12–23-month-old group than the 4.5–11-month-old group, suggesting delays in starting RI for older children, which is often linked to factors such as home delivery, migration, lack of awareness, or logistical barriers to accessing healthcare facilities [22]. The high prevalence of ZD children aligns with the findings from a study conducted by Biks and colleagues from Ethiopia, where a high proportion of ZD was noted as a result of regional disparities and systemic barriers; these were also associated with under-immunization [23]. These findings highlight the need for outreach programs to identify, enumerate, and vaccinate both cohorts’ (with special focus on older) children who may have missed their initial doses.

Caregiver education was significantly associated with delayed vaccination and ZD status in both cohorts. Logistic regression showed that higher educational levels were less likely to be associated with delayed or ZD vaccination status, aligning with the global trend that ties maternal education to better immunization uptake [22]. Educated caregivers are also more likely to comprehend the significance of vaccines, navigate healthcare systems, and adhere to schedules [24], while those with no formal education may lack the awareness of vaccine benefits or struggle with health literacy, hindering access [22,24]. These results suggest that boosting health education and literacy could significantly improve vaccination coverage in Kumbotso LGA. Furthermore, significant rural–urban disparities in ZD status among children aged 12–23 months emerged. Children in rural areas were nearly twice as likely to be ZD compared to their urban counterparts. Balogun et al. and Burroway & Hargrove found similar patterns from Nigeria, where not only maternal education but also residing in areas in which many women are educated influences the chances of having a ZD child [25,26]. This is also consistent with trends noted in other sub-Saharan African countries [22], where rural areas were often noted to face barriers, such as sparse healthcare infrastructure, longer travel distances, and lower socioeconomic status. Urban zones, conversely, often benefit from stronger healthcare access and outreach, likely contributing to fewer ZD cases [22].

In this study, while wealth quintiles showed significance in bivariate analysis, logistic regression revealed no independent effect on ZD status. This is in contrast with a study by Gichuki and colleagues from Kenya, where poverty strongly correlated with ZD status [27]. Furthermore, other studies from sub-Saharan Africa [22,28,29] also identified wealth as a key determinant of immunization uptake. Our finding suggests that other factors, such as education and settings, may mediate how socioeconomic status affects vaccination uptake. This may stem from the unique socioeconomic context of Kumbotso LGA, where even wealthier households may face other systemic barriers to immunization. The place of delivery was significantly associated with delayed vaccination and ZD status based on chi-square, though the logistic model did not validate this finding. Institutional deliveries are more likely to experience timely immunization, as healthcare providers can administer the first dose of vaccines shortly after birth and educate the caregivers about the schedule [29]. Expanding institutional deliveries through conditional cash transfers or non-financial incentives could improve immunization timeliness and reduce the number of ZD children in Kumbotso LGA. Caregiver employment showed no link with immunization outcomes, which contrasts with some studies linking maternal work conflicts as barriers [30]. However, it is worthy to note that there is a high proportion of self-employed caregivers from this study population, which may reflect the informal economy prevalent in northern Nigeria, where work schedules are more flexible, allowing caregivers to access immunization services.

About a quarter of the 12–23-month-old kids were ZD, signifying a high prevalence and necessitating the need for targeted outreach to identify and vaccinate the missed children. The gradual attrition in coverage from DPT1 to DPT3 (18%) highlights the challenges with follow-up and retention, as evidenced by dropout rates exceeding the national threshold value of 10% [1,31,32]. Strengthening defaulter tracking and community engagement strategies could improve retention and reduce dropout rates in Kumbotso LGA, as was successfully implemented in other similar settings [33]. BCG had the highest coverage, while birth-dose antigens like Hepatitis B had lower coverage. BCG coverage was highest, likely because BCG is administered up to 11 months [31], while the Hepatitis B birth dose lagged, reflecting high home delivery and facility practices to reduce vaccine wastage rate. Early education on institutional deliveries and other maternal health services could improve the coverage for birth-dose antigens [29].

This study approach identified three underperforming wards (Kumbotso, Unguwar Rimi, and Kureken Sani) with DPT3 coverage below 50%, consistent with Nigeria’s national dropout rate of 12–15% [2]. However, Challawa, Dan Maliki, Gurin Gawa, Mariri, Naibawa, and Panshekara wards demonstrated somewhat better performance relative to the LGA average. However, the vaccination rate for measles 1 was low, with only three [3] wards meeting the measles 1 target. These findings highlight the need to prioritize low-performing wards with high-impact interventions, such as Big Catchup, and tailored interventions to address ward-specific challenges [1]. These findings mirror those of the Zero Dose Situation Analysis [31] that reported similar challenges with measles vaccination in other parts of Nigeria, pointing to issues like vaccine availability, caregiver education, and health system weaknesses. The DIM approach’s ability to provide granular, ward-level data enables more precise targeting of resources and interventions.

The BeSD findings also revealed the central role of social and behavioral drivers in immunization uptake. Negative perceptions of vaccine importance and safety, coupled with distrust in health workers, were significant factors identified to be associated with delayed immunization and ZD status. These findings are consistent with the study from Mali that indicated that limited access to information, cultural norms, distrust in the health system, and logistical challenges contributed to poor immunization uptake among children [34]. Interestingly, caregivers who expressed positive vaccine intentions still had delayed or zero-dose children, suggesting a gap between intent and action, often due to structural barriers like stockouts, disrespectful treatment at facilities, or lack of spousal approval.

Gender dynamics’ role was evident, with nearly all caregivers needing spousal or familial approval to vaccinate, reflecting the patriarchal norm in northern Nigeria [35]. Addressing power structures through community and religious leaders and addressing gender-sensitive strategies could improve uptake. While caregiver intent to vaccinate strongly predicted timely immunization, many with positive intentions still faced delays or ZD status, suggesting systemic barriers such as vaccine stockouts or healthcare access issues [30]. Bridging this intent–action divide requires strengthening the health systems and incentives to align caregiver willingness with actionable pathways. Community-based interventions involving religious leaders could mitigate this barrier.

Most delayed/ZD caregivers spend under 200 Naira (~USD 0.15) on transport, suggesting proximity is not the main barrier. Instead, cultural norms and low vaccine awareness—not access—drive low uptake in Kumbotso LGA. Culturally tailored education and community engagement could address these gaps [1]. This finding suggests that proximity is not a major barrier in Kumbotso LGA. Instead, poor service quality, provider attitudes, and low awareness are more salient issues, echoing findings from a systematic review conducted in sub-Saharan Africa by Bangura and colleagues to uncover factors that hinder childhood immunization, where caregiver-related barriers were more pronounced than those of health care providers and health systems [36]. High card retention rates from this study among vaccinated children point to an opportunity to strengthen follow-up through improved appointment scheduling and reminder systems. While most caregivers knew where to vaccinate and found services affordable, dissatisfaction (long waits, disrespectful treatment) hindered uptake, especially for 12–23-month-olds. This also aligns with evidence that poor service quality undermines immunization [22]. Improving service quality through training healthcare workers and reducing wait times could enhance caregiver satisfaction and vaccination uptake.

This study aligns with the measure and monitor component of the IRMMA framework adopted by Nigeria’s ZD CLH objectives to generate evidence on effective strategies in tracking ZD reduction [8]. This study also established a baseline for monitoring progress in Kumbotso towards the Immunization Agenda 2030 (IA2030) and Gavi’s 5.0 guidelines [4,37], advancing strategies to close the immunization gaps through localized, evidence-driven action.

### 5.1. Limitations

The exclusion of newly relocated residents and secondary caregivers may have led to underrepresentation of transient or marginalized populations. Future studies should explore strategies to include these groups to ensure equal representation. Furthermore, the study utilized recall from primary caregivers when cards were unavailable; this may have introduced information bias, particularly in the reporting of vaccination status for the 12–23-month-old birth cohort. Finally, the cross-sectional design of the study restricts causal inference, limiting the ability to establish temporal relationships between predictors and vaccination outcomes in both age cohorts. Future studies should consider longitudinal designs and integrate qualitative inquiry to provide a deeper understanding of ZD dynamics.

### 5.2. Recommendations

Based on the findings from the pilot study of the DIM project in Kumbotso Local Government, Kano State, we came up with several recommendations outlined below:

1. Scale up DIM in other LGAs with high rates of ZD children to prioritize resource allocation and tailored interventions (e.g., DPT/measles catch-up campaigns) in low-coverage wards identified via LQAS.
2. Address BeSD-identified barriers (limited female autonomy, healthcare worker distrust, safety concerns) through community engagement (religious leaders, women’s groups, dialogues) to counter misinformation.
3. Train healthcare workers in communication/client-centered counseling, and implement mentorship programs to build caregiver trust in vaccination.
4. Conduct biannual DIM monitoring to track zero-dose reduction, identify challenges, and guide RI program adjustments.
5. Ensure DIM data utilization by government/stakeholders for informed decision-making, resource allocation, and program improvement.

## 6. Conclusions

The pilot application of DIM in Kumbotso LGA demonstrates its potential as an important tool for tracking the reduction in the number of zero-dose kids and for the decision-making process across LGA, state, and national levels. The identification of sociocultural, geographic, and service delivery barriers to vaccination using the DIM approach provides a new method for improving routine immunization coverage systematically by the use of data for action. Scaling up this approach across other LGAs in Nigeria is necessary to achieving national and global immunization targets and thereby reducing the burden of vaccine-preventable diseases and advancing equity in health care delivery.

## Figures and Tables

**Table 1:** Demographic characteristics of sampled caregivers.

Characteristics	Overall, N = 418
**Age of Caregiver (years)**	28 (SD ± 6.2)
**Sex of Eligible Children Sampled**	
Male	206 (49.3%)
Female	212 (50.7%)
**Setting**	
Rural	103 (24.6%)
Urban	315 (75.4%)
**Religion**	
Christian	9 (2.8%)
Islam	409 (97.9%)
**Caregivers’ Marital Status**	
Married	415 (99.3%)
Divorced	2 (0.5%)
Widow	1 (0.2%)
**Educational Qualification **	
No Formal Education	147 (35.2%)
Primary School Cert	44 (10.5%)
Senior Secondary School Cert	186 (44.50%)
Post-Secondary School	41 (9.81%)
**Employment Status**	
Self-Employed	277 (66.27%)
Not engaged/no source of income	103 (24.64%)
Formally employed	38 (9.09%)
**Wealth Quintile **	
Quintile 1	71 (17.46%)
Quintile 2	112 (26.79%)
Quintile 3	116 (27.75%)
Quintile 4	74 (17.70%)
Quintile 5	43 (10.29%)
**Eligible Child Age Categorization **	
<4.5 Months	81 (19.4%)
4.5–11 Months	128 (30.6%)
12–23 Months	209 (50.0%)

**Table 2:** Socioeconomic and demographic factors affecting DPT uptake using Chi square test.

	4.5 to 11 Months (n = 128)			12 to 23 Months (n = 209)		
	Delayed	Undelayed	*p*-Value	Zero Dose	Non-Zero Dose	*p*-Value
Characteristics	No DPT (n = 28)	DPT (n = 100)		No DPT (n = 56)	DPT (n = 153)	
Caregiver Age (Mean ± SD)	29.2 ± 5.9	28.9 ± 5.8	0.840	28.8 ± 6.9	28.9 ± 6.0	0.930
Religion			0.451			0.613
Islam	28 (100.0)	98 (98.0)		56 (100.0)	150 (98.0)	
Christianity	0 (0.0)	2 (2.0)		0 (0.0)	3 (2.0)	
Setting			0.472			<0.001 *
Rural	8 (28.6)	22 (22.0)		28 (50.0)	25 (16.3)	
Urban	20 (71.4)	78 (78.0)		28 (50.0)	128 (83.6)	
Educational status of caregiver			0.003 *			<0.001 *
No formal Education	19 (67.8)	28 (28.0)		39 (70.0)	38 (24.8)	
Primary Education	3 (10.7)	13 (13.0)		3 (5.4)	21 (13.7)	
Secondary Education	6 (21.4)	49 (49.0)		13 (23.1)	73 (47.7)	
Tertiary Education	0 (0.0)	10 (10.0)		1 (1.8)	21 (13.7)	
Employment status of caregiver			0.214			0.204
Not engaged/No income source	6 (21.4)	27 (27.0)		14 (25.0)	34 (22.0)	
Self-employed	22 (78.5)	65 (65.0)		40 (71.4)	101 (66.0)	
Formally employed	0 (0.0)	8 (8.0)		2 (3.6)	18 (12.0)	
Wealth Quintile			0.034 *			0.010 *
Quintile 1	6 (21.4)	16 (16.0)		16 (28.6)	24 (15.7)	
Quintile 2	13 (46.4)	25 (25.0)		18(31.1)	32 (20.9)	
Quintile 3	7 (25.0)	24 (24.0)		14 (25.0)	46 (30.1)	
Quintile 4	2 (7.1)	23 (23.0)		7 (12.5)	27 (17.7)	
Quintile 5	0 (0.0)	12 (12.0)		1 (1.8)	24 (15.7)	
Place of delivery			0.008 *			<0.001 *
Traditional Birth Attendant	1(3.6)	1 (1.0)		3 (5.4)	8 (5.2)	
Home	21 (75.0)	44 (44.0)		43 (77.0)	58 (38.0)	
Private facility	0 (0.0)	7 (7.0)		0 (0.0)	16 (10.0)	
Government facility	5 (17.9)	46 (46.0)		9 (16.0)	68 (44.0)	
Others	1 (3.6)	2 (2.0)		1 (1.8)	3 (2.0)	

* Statistically significant.

**Table 3:** Logistic regression of factors associated with delayed vaccination among 4.5–11-month age group.

Factor	Coefficient	Odd Ratio	95% CI	*p*-Value	
Educational level	−1.08149	0.34	0.22–0.53	<0.0001	*
Wealth index	−0.129208	0.88	0.71–1.09	0.234	
Place of delivery	−0.234010	0.79	0.54–1.16	0.229	

* Statistically significant.

**Table 4:** Sociodemographic factors associated with zero dose using logistic regression for the 12-to-23-month age group.

Factor	Coefficient	Odd Ratio	95% CI	*p*-Value	
Caregiver educational level	−0.980558	0.37	0.24–0.58	<0.0001	*
Wealth index	−0.142458	0.87	0.70–1.07	0.197	
Place of delivery	−0.159415	0.85	0.58–1.25	0.415	
Setting	0.637404	1.89	1.04–3.44	0.036	*

* Statistically significant.

**Table 5:** Antigen-specific analysis for children aged 12–23 months.

RI Antigens	Frequency—Unweighted (N = 209)	Weighted % Coverages	95% CI
BCG	167 (79.9%)	81.5%	81.0–81.9%
HepB 0	137 (65.6%)	71.2%	70.8–71.6%
OPV 0	157 (75.1%)	77.9%	77.5–78.3%
DPT 1	153 (73.2%)	75.5%	75.1–75.9%
DPT 2	137 (65.6%)	66.6%	66.2–67.0%
DPT 3	128 (61.2%)	63.6%	63.2–64.0%
IPV 1	145 (69.4%)	72.3%	71.9–72.7%
IPV 2	117 (56.0%)	60.0%	56.9–60.4%
PCV 1	154 (73.7%)	75.7%	75.3–76.1%
PCV 2	140 (67.0%)	68.2%	67.8–68.6%
PCV 3	129 (61.7%)	64.0%	63.5–64.4%
Measles 1	120 (57.4%)	59.7%	59.3–60.1%

**Table 6:** BeSD indicators categorized by receipt of DPT1 or not and by age group.

Characteristics	4.5–11 Months N = 128			12–23 Months N = 209		
	Delayed, N = 28	Undelayed, N = 100	*p*-Value (0.05)	ZD, N = 56	NZD, N = 153	*p*-Value (0.05)
Vaccine Importance			<0.001 *			<0.001 *
Negative	14 (50.0%)	3 (3.0%)		30 (53.6%)	7 (4.6%)	
Positive	14 (50.0%)	97 (97.0%)		26 (46.4%)	146 (95.4%)	
Vaccine Safety			<0.001 *			<0.001 *
Negative	13 (46.4%)	4 (4.0%)		28 (50.0%)	6 (3.9%)	
Positive	15 (53.6%)	96 (96.0%)		28 (50.0%)	147 (96.1%)	
Healthcare Workers Trust			<0.001 *			<0.001 *
Little or No Trust in HCWs	13 (46.4%)	4 (4.0%)		27 (48.2%)	8 (5.2%)	
Trust HCWs	15 (53.6%)	96 (96.0%)		29 (51.8%)	145 (94.8%)	
Caregiver Intention to Vaccinate			<0.001 *			<0.001 *
None	6 (21.4%)	2 (2.0%)		18 (32.1%)	2 (1.3%)	
Some	15 (53.6%)	10 (10.0%)		21 (37.5%)	21 (13.7%)	
All	7 (25.0%)	88 (88.0%)		17 (30.4%)	130 (85.0%)	
Service Satisfaction			0.332			0.047 *
Satisfied	7 (87.5%)	97 (97.0%)		12 (80.0%)	146 (95.4%)	
Unsatisfied	1 (12.5%)	3 (3.0%)		3 (20.0%)	7 (4.6%)	
Ease of Vaccination			0.142			<0.001 *
Affordable	21 (75.0%)	87 (87.0%)		31 (55.4%)	132 (86.3%)	
Unaffordable	7 (25.0%)	13 (13.0%)		25 (44.6%)	21 (13.7%)	
Knowledge of Where to get Child Vaccinated	28 (100.0%)	100 (100.0%)	<0.001 *	49 (87.5%)	152 (99.3%)	<0.001 *
Religious Leader Supports Vaccination.	26 (92.9%)	96 (96.0%)	0.601	52 (92.9%)	150 (98.0%)	0.085
Community Leader supports vaccination.	26 (92.9%)	97 (97.0%)	0.321	54 (96.4%)	148 (96.7%)	>0.914
Friends and Close Family Members support vaccination.	22 (78.6%)	96 (96.0%)	0.007 *	45 (80.4%)	151 (98.7%)	<0.001 *
Parents support vaccination (Peer Norm)	24 (85.7%)	97 (97.0%)	0.041 *	48 (85.7%)	143 (93.5%)	0.100
Need permission to vaccinate	26 (92.9%)	99 (99.0%)	0.120	52 (92.9%)	152 (99.3%)	0.19 *

* Statistically significant.

## Data Availability

The dataset generated and analyzed in the current study are not publicly available due to data use restrictions but are available from the corresponding author upon reasonable request. Access will require a formal Data Use Agreement. Data generated are store securely and will be provided with codebook to aid interpretation. For access contact the corresponding author.

## Abbreviations

The following abbreviations are used in this manuscript:

BeSD	Behavioural and Social Drivers of Vaccination
BCG	Bacille Calmette–Guérin
CA	Catchment Area
CLH	Country Learning Hub
DIM	Decentralized Immunization Monitoring
DPT	Diphtheria, Pertussis, and Tetanus
HREC	Health Research and Ethics Committee
HH	Household
IPV	Inactivated Polio Vaccine
IRMMA	Identify–Reach–Monitor–Measure–Advocacy
LGA	Local Government Area
LQAS	Lot Quality Assurance Sampling
NDHS	National Demographic and Health Survey
NZD	Non-Zero-Dose Children
OPV	Oral Polio Vaccine
PCV	Pneumococcal Conjugate Vaccine
RI	Routine Immunization
RITWG	Routine Immunization Technical Working Group
SA	Supervision Area
SPHCMB	State Primary Health Care Management Board
ZD	Zero-Dose Children
